# ‘Fit for school’ – a school-based water, sanitation and hygiene programme to improve child health: Results from a longitudinal study in Cambodia, Indonesia and Lao PDR

**DOI:** 10.1186/s12889-017-4203-1

**Published:** 2017-04-05

**Authors:** Denise Duijster, Bella Monse, Jed Dimaisip-Nabuab, Pantjawidi Djuharnoko, Roswitha Heinrich-Weltzien, Martin Hobdell, Katrin Kromeyer-Hauschild, Yung Kunthearith, Maria Carmela Mijares-Majini, Nicole Siegmund, Panith Soukhanouvong, Habib Benzian

**Affiliations:** 1 Department of Social Dentistry, Academic Centre for Dentistry Amsterdam, Gustav Mahlerlaan 3004, 1081LA Amsterdam, The Netherlands; 2 Department of Epidemiology and Public Health, University College London, Torrington Place 1-19, London, WC1E 6BT UK; 3 Gesellschaft für Internationale Zusammenarbeit (GIZ), L.P. Leviste cor Rufino Street, Makati City, Metro Manila, Philippines; 4 Social Basic Services Bureau of West Java, Governor’s Office, Jl. Diponegoro No. 22, Citarum, Bandung Wetan, Bandung, West Java 40115 Indonesia; 5 Department of Preventive Dentistry and Pediatric Dentistry, University Hospital Jena, Friedrich Schiller University Jena, Bachstraße 18, 07743 Jena, Germany; 6 Institute of Human Genetics, University Hospital Jena, Friedrich Schiller University Jena, Kollegiengasse 10, 07740 Jena, Germany; 7 Department of School Health of the Ministry of Education, Youth and Sport of the Kingdom of Cambodia, Street 380, Chao Ponheahok Primary School, BKK1, Khann Chamkarmorn, Phnom Penh, Cambodia; 8 Department of Preschool and Primary Education, Ministry of Education and Sports, Ministry of Education Building No 1, Lane Xang Ave, P.O. Box 067, Vientiane Capital, Laos; 9 Department of Epidemiology and Health Promotion, College of Dentistry, New York University, 433 First Avenue, New York, NY 10010 USA

**Keywords:** School health, Water sanitation and hygiene, Handwashing, Toothbrushing, Deworming, Dental caries, Underweight, Soil-transmitted helminth infection

## Abstract

**Background:**

The Fit for School (FIT) programme integrates school health and Water, Sanitation and Hygiene interventions, which are implemented by the Ministries of Education in four Southeast Asian countries. This paper describes the findings of a Health Outcome Study, which aimed to assess the two-year effect of the FIT programme on the parasitological, weight, and oral health status of children attending schools implementing the programme in Cambodia, Indonesia and Lao PDR.

**Methods:**

The study was a non-randomized clustered controlled trial with a follow-up period of two years. The intervention group consisted of children attending public elementary schools implementing the FIT programme, including daily group handwashing with soap and toothbrushing with fluoride toothpaste, biannual school-based deworming; as well as construction of group handwashing facilities. Control schools implemented the regular government health education curriculum and biannual deworming. Per school, a random selection of six to seven-year-old grade-one students was drawn. Data on parasitological infections, anthropometric measurements, dental caries, odontogenic infections and sociodemographic characteristics were collected at baseline and at follow-up (24 months later). Data were analysed using the χ^2^-test, Mann Whitney U-test and multilevel logistic and linear regression.

**Results:**

A total of 1847 children (mean age = 6.7 years, range 6.0–8.0 years) participated in the baseline survey. Of these, 1499 children were available for follow-up examination – 478, 486 and 535 children in Cambodia, Indonesia and Lao PDR, respectively. In all three countries, children in intervention schools had a lower increment in the number of decayed, missing and filled permanent teeth between baseline and follow-up, in comparison to children in controls schools. The preventive fraction was 24% at average. The prevalence of soil-transmitted helminth infection (which was unexpectedly low at baseline), the prevalence of thinness and the prevalence of odontogenic infections did not significantly differ between baseline and follow-up, nor between intervention and control schools.

**Conclusions:**

The study found that the FIT programme significantly contributed to the prevention of dental caries in children. This study describes the challenges, learnings and, moreover, the importance of conducting real-life implementation research to evaluate health programmes to transform school settings into healthy learning environments for children.

The study is retrospectively registered with the German Clinical Trials Register, University of Freiburg (Trial registration number: DRKS00004485, date of registration: 26th of February, 2013).

## Background

Improvements of Water, Sanitation and Hygiene (WASH) are fundamental to promote child health in low and middle-income countries. Water scarcity, limited access to improved sanitation and lack of personal hygiene at home and in school significantly contribute to the immense burden of preventable childhood diseases, such as diarrhoea, acute respiratory infections, intestinal worms and dental caries [1–3]. These hygiene-related illnesses add to a vicious cycle of poverty and disease through their adverse impacts on children’s school attendance, educational performance and productivity [4–6].

Improving WASH in Schools (WinS) is a key intervention to increase children’s prospects for a healthy development [7]. It contributes to a safe and healthy learning environment and is a prerequisite for teachers and students to develop and practice positive hygiene habits. WinS has gained increasing attention on political agendas, particularly in the development sector, as evidenced by the inclusion of WinS targets and respective indicators as part of the Sustainable Development Goals (SDGs) [8]. The growth of the WinS sector is also visible in the area of research. The benefit of school-based handwashing with soap is now well established; this intervention alone has been shown to prevent around one-third of diarrhoea episodes in children [9]. There is promising evidence from a few recent cluster-randomized trials that WinS programmes, such as school-based hygiene promotion, water treatment and improved sanitation, are effective in reducing pupil absenteeism by 21% to 58%, in some cases specifically for girls [10–13]. However, there are not many studies evaluating the benefits of WinS programmes on children’s health outcomes [7], which are generally challenging to measure. Strong evidence for such health effects, together with research on best methods of integrating WASH into school health programmes, would allow for stronger advocacy and foster the adoption of appropriate WASH policies within the education sector.

The Fit for School (FIT) approach is an integrated school health promotion and WinS concept, which has been developed as a response to the serious health problems of Southeast Asian children [14]. The FIT approach aims to improve child health through the institutionalization of a combination of simple, evidence-based preventive interventions, including improvement of WASH facilities, daily practice of group hygiene activities and school-based deworming. The implementation of the FIT approach is conceptually based on the principles of ‘simplicity’, ‘scalability’, ‘sustainability’ and ‘system-thinking’ [15], which address the concepts of intersectoral collaboration, sustainable financing mechanisms, active community involvement and the strengthening of school-based management. The FIT approach originated in the Philippines in 2007/2008 where it was implemented by the Department of Education as the ‘Essential Health Care Programme’ [14]. In 2011, commissioned by the German Federal Ministry for Economic Cooperation and Development, the Deutsche Gesellschaft für Internationale Zusammenarbeit (GIZ) and the South-East Asian Ministers of Education Organization Regional Centre for Educational Innovation and Technology (SEAMEO INNOTECH) partnered to expand the FIT approach to Cambodia, Indonesia and Lao PDR as the ‘Regional Fit for School Programme’. The Regional FIT Programme supported the respective Ministries of Education (MoEs) to adapt the concept to local conditions and start implementation in model schools during a pilot phase from 2012 to 2014.

**Fig. 1:**
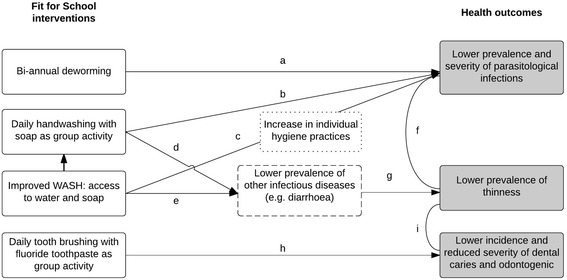
Hypothesised health outcomes of the Fit for School programme, based on available evidence. *Grey boxes* represent hypothesised health outcomes resulting from the FIT programme interventions. White boxes with dashed lines represent intermediate health outcomes that were not assessed in this study. White boxes with dotted lines represent intermediate behavioural outcomes that were not assessed in this study. Summary of related evidence: **a** Biannual deworming reduces the prevalence and severity of intestinal worm infection [28]. **b, c, d, e** Handwashing with soap and improved access to WASH have been associated with lower prevalence of STH infections [25, 29] and other infectious diseases, such as diarrhoea [10, 30]. **f, g** Lower prevalence of worm infection and diarrhoea have been associated with weight gain [35, 46]. **h** Toothbrushing with fluoride toothpaste prevents dental caries and odontogenic infections [31]. **i** Lower prevalence of dental caries and odontogenic infections are associated with lower prevalence of thinness [38] and weight gain [39]

The piloting of the programme was accompanied by an extensive *Fit for School Programme Assessment Study*, which comprised three study components - a WASH survey, a behaviour study and a Health Outcome Study (HOS). This paper describes the findings of the HOS, which aimed to assess the two-year-effect of the FIT interventions on the parasitological, weight and oral health status of children attending schools implementing the FIT programme in Cambodia, Indonesia and Lao PDR. The hypothesised health outcomes of the study, supported by available evidence, are presented in Fig. 1. The findings of the WASH survey and the behaviour study will be reported in separate papers.

## Methods

### Study design

The study was designed as a non-randomized clustered controlled trial. It describes a longitudinal cohort of children that were followed-up for a period of two years. The intervention group consisted of public elementary schools implementing the FIT programme interventions, including:

- Daily handwashing with soap as a group activity,
- Daily toothbrushing with 0.3 ml of toothpaste (containing 1450 ppm free available fluoride) as a group activity,
- Biannual deworming with a single dose of albendazole or mebendazole (400 mg tablet) as part of the respective national government-coordinated deworming programme.

Access to water and soap is a prerequisite for the practice of these daily hygiene activities. Therefore, the FIT programme supported the construction and maintenance of group washing facilities, which serve as a starting point for stepwise improvement of other aspects of WinS, such as availability of appropriate sanitation facilities. Group washing facilities consisted of prefabricated facilities containing several water slots to accommodate many students for group hygiene activities [16]. Educational staff in the intervention schools received practical guidance and training materials, but no further support to implement the programme activities. The control group included public elementary schools that implemented nothing else than the regular government health education curriculum and biannual deworming as part of the national deworming programme. The national deworming programme has been implemented since 2004, 1999, and 2005 in Cambodia, Indonesia and Lao PDR respectively. Baseline data were collected in 2012 - two weeks before the implementation of the FIT programme - and follow-up data were collected 24 months later in 2014.

The study’s original methodology and protocol was developed in the Philippines in 2009 [17]. The HOS in Cambodia, Indonesia and Lao PDR followed a similar methodology.

### Study sample and procedure

The study included a total of 41 intervention schools implementing the FIT programme: 10 schools in Cambodia (Pnomh Penh, and the provinces Kampot, Takeo, Kampong Thom and Kampong Chhnang), 9 schools in Indonesia (Bandung City and Indramayu) and 22 schools in Lao PDR (Vientiane Capital and surroundings). Selection of the intervention schools was done by the respective MoEs on the basis of accessibility and support from the school administration. For each intervention school, the nearest public elementary school with the same classification according to the size of the school (school population) was assigned as a control school.

A power calculation indicated that samples of 600 children per country (300 children per group) were required. The sample size was based upon detecting a 20% difference in mean caries increment between intervention and control schools after a 24-month period [17] with a statistical power of 80% and a significance level of 5%. This would also provide adequate power to detect a 15% difference in the proportion of thin children and children with soil-transmitted helminth (STH) infection. The sample size was increased to 720 children per country to allow for a drop-out rate of 20% without compromising on the statistical power.

Per school, a random selection of 36, 40 and 17 six- to seven-year-old children was drawn from the list of enrolled grade-one students for the baseline study in Cambodia, Indonesia and Lao PDR, respectively. The same children were re-examined after 24 months. Consent for their participation was secured from the parents or guardians by school representatives. Children with no parental or guardian consent were excluded from the study.

### Data collection

In each country, data collection was performed by a team of local researchers from partner institutions, including the MoEs, the oral health and health offices of the Ministries of Health (MoHs), the Faculty of Dentistry of Universitas Padjadjaran in Indonesia and the University of Health Sciences in Lao PDR. Prior to data collection, examiners participated in a three-day training on standardised data collection methods and a calibration process. All examiners were blind to which group the schools belonged.

Data collection took place on the school ground following a standard procedure: registration and stool specimen collection, anthropometric measurement, oral examination and a socio-demographic interview.

#### Parasitological examination

Children submitted a stool sample on the day of data collection. Within the same day, labelled stool specimens were brought to the MoH Centre for Malaria, Parasitology and Entomology laboratory in Lao PDR and the West Java Provincial Health Laboratory and the Indramayu District Health Laboratory in Indonesia for examination. In Cambodia stool specimens were directly examined on the school ground by staff from the MoH Centre for Malaria, Parasitology and Entomology laboratory. Samples were examined to determine the presence and intensity of STH infection (*Ascaris* species, hookworm and *Trichuris* species) using the Kato-Katz method [18]. Cut-off points defined by the WHO were used to classify light-, moderate-, and heavy-intensity infections [19]. Ten percent of stool samples were re-examined by a reference microscopist for quality control.

#### Anthropometric measurement

Children’s weight and height were measured in duplicate following standards described by Cogill [20] The average of two measurements was recorded. A SECA digital weighing scale (calibrated at the beginning of each day and after every 10th child) was used to measure weight to the nearest 0.1 kg. Standing height was measured to the nearest 0.1 cm using a microtoise. Body mass index (BMI) was computed as weigh/height^2^ (kg/m^2^) and converted to BMI for age *z*-scores using the 2007 WHO Growth reference for school-aged children and adolescents [21]. Thinness and overweight were defined as a BMI for age below and above 2SDs from the WHO growth reference median, respectively [22].

#### Oral examination

In each country, four calibrated dentists performed oral examinations in the schoolyard or inside a classroom to collect data on oral health status. Oral health status referred to dental caries experience and the presence of odontogenic infections, which are the two most common oral diseases among children. Children were placed in supine position on a classroom bench, table or series of chairs with their heads on a pillow placed on the lap of the dentist. Mouth mirrors with illumination (Mirrorlite) and a CPI-ball-end probe were used to score dental caries according to WHO Basic Methods for Oral Health Surveys [23]. Dental caries experience was expressed as the dmft/DMFT-index by calculating the sum of decayed (d/D), missing (m/M) and filled (f/F) teeth (t/T). Odontogenic infections were scored according to the criteria of the pufa/PUFA-index [24], which records the presence of open pulp (p/P), ulceration (u/U), fistula (f/F) and abscesses (a/A). For both indexes, uppercase letters indicate the permanent dentition, and lowercase letters indicate the primary dentition. Kappa-scores for inter-examiner reliability ranged from 0.73 to 0.97 (mean *k* = 0.87) for dmft/DMFT and from 0.58 to 1.00 (mean *k* = 0.78) for pufa/PUFA.

#### Covariates

All children completed an interview-questionnaire in native language to collect demographic information, including date of birth and gender. Socioeconomic status (SES) was assessed using six questions as proxy-indicators: ‘Do you have a TV at home?’ (Yes/No), ‘Do you have a car at home?’ (Yes/No), How many brothers do you have?’, ‘How many sisters do you have?’, ‘Did you eat breakfast today?’ (Yes/No) and ‘Did you eat lunch yesterday?’ (Yes/No). Only the number of siblings (family size) was used as an indicator of SES in this study, since the other variables showed little variance. Children were also asked about the presence of mouth problems and abdominal pain at the time of examination.

Data from the WASH survey were used to obtain information on school characteristics, including the number of enrolees per school and the number of handwashing facilities with water and soap available. In addition, data on sanitation were collected, including the number, functionality and cleanliness of toilets, as proxy indicators of school maintenance and cleanliness in general, and as a covariate since there is evidence that access and cleanliness of sanitation facilities influences children’s parasitological health [25]. Data in the WASH survey were collected at baseline and follow-up through observations using an adapted version of the UNICEF WASH in Schools Monitoring Observational Tool [26]. Per country, two researchers of the local research team were trained to conduct the WASH survey in the schools. Toilets were scored as clean, partially clean or not clean, and as functional, partially functional and not functional. Toilets were subsequently classified as both clean and functional, partially clean and/or functional, or not clean and/or functional. Data from the WASH survey were solely used to describe the schools in the study sample, and for inclusion as potentially important covariates in the analysis of children’s health outcomes. The full findings of the WASH survey will be reported in a separate paper.

### Statistical analysis

Data were analyzed using STATA 13 (Stata Corp, College Station, Texas, USA). A *P*-value of <0.05 was regarded as significant. Complete case analysis was used to handle missing data.

Differences in health outcomes between intervention and control schools were analyzed for each country separately, and for the overall sample. The χ^2^-test was used to assess differences in the prevalence of STH infection, the prevalence of thinness and the prevalence of dental caries and odontogenic infections (all expressed in percentages); the Mann Whitney U-test was used to assess differences in the mean DMFT increment and PUFA increment. The preventive fraction for DMFT was calculated, which is the difference in mean DMFT increment between the intervention and control schools expressed as a percentage of the mean DMFT increment in the control group. Since existing dental caries lesions cannot disappear or decrease through intervention, and primary teeth are exfoliating at the age of children’s examination, the analysis of caries progression and odontogenic infection was limited to the permanent dentition only.

Furthermore, multilevel logistic and linear regression analyses with backward selection were performed to explore which factors (including the FIT programme) were associated with STH infection at follow-up (no infection vs infection), thinness at follow-up (normal weight or overweight vs thinness) and DMFT increment between baseline and follow-up (continuous). Variables considered in the models were the FIT programme (control schools vs. intervention schools), sociodemographic characteristics and health parameters of the children, and school characteristics. Because children (first level) were nested in schools (second level), which were in turn nested in the three countries (third level), multilevel analyses were used to control for the possible effect of clustered differences within the sample. For each model, the intraclass correlation coefficient (ICC) was calculated to indicate the percentage of total variance that was due to differences between schools or the countries.

In eight schools in Lao PDR (four intervention and four control schools), the simultaneous implementation of an oral health programme from the Japanese International Cooperation Agency, providing dental treatment (restorations) and fluoride rinsing, interfered with the implementation of the Fit for School programme without knowledge of the study investigators. During the follow-up data collection the fact was revealed and it was decided to exclude these eight schools from the analysis of oral health outcomes.

### Ethical approval

The study received ethical approval from the National Ethics Committee for Health Research of the MoHs in Cambodia and Lao PDR, and from the Health Research Ethics Committee of the University of Padjadjaran, Indonesia. The study is registered with the German Clinical Trials Register, University of Freiburg (Trial registration number: DRKS00004485, date of registration: 26th of February, 2013). Parents of participating children provided written informed consent.

## Results

### Characteristics of the study sample

A total of 1847 children participated in the baseline survey. Of these, 1499 children were available for follow-up examination – 478 children in Cambodia (241 in intervention schools and 237 in control schools), 486 children in Indonesia (248 in intervention schools and 238 in control schools) and 535 children in Lao PDR (279 in intervention schools and 256 in control schools). The follow-up rate was 76.6%, 85.3% and 81.0% in Cambodia, Indonesia and Lao PDR, respectively, with an average follow-up rate of 81.2%. Parasitological, anthropometric and oral health parameters of the dropout children were similar to those children who were followed-up. The mean time interval between baseline and follow-up was 23.9 ± 0.3 months.

The child characteristics of the study sample are described in Table 1. The mean age of the children at baseline was 6.7 ± 0.5 years (range 6.0–8.0 years) in intervention schools and 6.8 ± 0.5 years (range 6.0–8.0 years) in control schools (*P* < 0.05), and 48.4% and 53.9% were boys in intervention and control schools, respectively (*P* < 0.05). Around one-third of children came from large families with three or more siblings – a proxy indicator of lower SES.

**Table 1:** Child characteristics of the study sample in Cambodia, Indonesia, Lao PDR and the pooled regional countries

	Cambodia			Indonesia			Lao PDR			Regional (pooled)		
	FIT (*n* = 241)	Control (*n* = 237)		FIT (*n* = 248)	Control (*n* = 238)		FIT (*n* = 279)	Control (*n* = 256)		FIT (*n* = 768)	Control (*n* = 731)	
Child characteristics	*mean ± sd*	*mean ± sd*	P*	*mean ± sd*	*mean ± sd*	P*	*mean ± sd*	*mean ± sd*	P*	*mean ± sd*	*mean ± sd*	P*
Age at baseline	6.6 ± 0.4	6.7 ± 0.5	0.143	6.8 ± 0.4	6.8 ± 0.4	0.129	6.7 ± 0.5	6.8 ± 0.6	*0.026*	6.7 ± 0.5	6.8 ± 0.5	*0.003*
	*n (%)*	*n (%)*		*n (%)*	*n (%)*		*n (%)*	*n (%)*		*n (%)*	*n (%)*	
Gender			0.780			0.100			0.088			*0.034*
Boys	122 (50.6)	123 (51.9)		118 (47.6)	131 (55.0)		132 (47.3)	140 (54.7)		372 (48.4)	394 (53.9)	
Girls	119 (49.4)	114 (48.1)		130 (52.4)	107 (45.0)		147 (52.7)	116 (45.3)		396 (51.6)	337 (46.1)	
Family size^a, b^			0.115			0.350			0.751			0.166
1 or no siblings	83 (34.4)	61 (25.7)		131 (53.0)	122 (51.5)		108 (38.7)	91 (35.6)		322 (42.0)	274 (37.5)	
2 siblings	64 (26.6)	73 (30.8)		77 (31.2)	66 (27.9)		95 (34.1)	92 (35.9)		236 (30.8)	231 (31.6)	
3 or more siblings	94 (39.0)	103 (43.5)		39 (15.8)	49 (20.7)		76 (27.2)	73 (28.5)		209 (27.2)	225 (30.8)	

* χ^2^-test

^a^ Measured at follow-up

^b^ Missing values: Cambodia: 0, Indonesia: 2, Lao PDR: 1

Table 2 describes the characteristics of the intervention and control schools. All schools in Indonesia were located in urban areas; Cambodia and Lao PDR also included schools in rural provinces. In all three countries, the mean number of handwashing slots with water and soap was significantly higher in intervention schools than in control schools, due to the construction of group washing facilities as part of the programme implementation [27]. Consequently, substantially less children had to share one water slot in intervention schools compared to the control schools (Table 2). Access to toilets was similar in intervention and control schools. However, toilet conditions in terms of functionality and cleanliness were slightly better in intervention schools, although this was only statistically significant for Lao PDR and the overall sample.

**Table 2:** Characteristics of the schools in the study sample in Cambodia, Indonesia, Lao PDR and the pooled regional countries

	Cambodia			Indonesia			Lao PDR			Regional (pooled)		
School characteristics^**a**^	FIT (*n* = 10)	Control (*n* = 10)	P*	FIT (*n* = 9)	Control (*n* = 9)	P*	FIT (*n* = 22)	Control (*n* = 22)	P*	FIT (*n* = 41)	Control (*n* = 41)	P*
Geographical location *(n)*												
Rural school	6	6		-	-		7	7		13	13	
Urban school	4	4		9	9		15	15		28	28	
No. of enrollees *(mean n ± sd)*	805 ± 384	723 ± 228	0.999	592 ± 323	388 ± 220	0.200	219 ± 124	118 ± 64	*0.003*	505 ± 346	391 ± 295	*0.046*
No. of handwashing slots w/ water & soap *(mean n ± sd)*	200 ± 128	9 ± 16	*0.001*	89 ± 57	1 ± 2	*<0.001*	113 ± 69	17 ± 38	*<0.001*	129 ± 93	11 ± 30	*<0.001*
Student to handwashing slot ratio *(mean ratio ± sd)*	4:1 ± 1:1	55:1 ± 46:1	*<0.001*	6:1 ± 1:1	74:1 ± 71:1	*<0.001*	2:1 ± 2:1	66:1 ± 63:1	*0.001*	4:1 ± 2:1	65:1 ± 60:1	*<0.001*
Student to toilet ratio *(mean ratio ± sd)*	93:1 ± 56:1	102:1 ± 53:1	0.496	99:1 ± 50:1	112:1 ± 61:1	0.691	63:1 ± 44:1	45:1 ± 24:1	0.218	79:1 ± 50:1	74:1 ± 52:1	0.593
Percentage of clean & functional toilets *(mean % ± sd)*	7.6 ± 14.0	0.0 ± 0.0	0.068	62.2 ± 41.8	36.1 ± 39.7	0.219	37.6 ± 44.1	15.9 ± 35.9	*0.026*	35.6 ± 42.0	16.5 ± 33.8	*0.007*

*Mann Whitney U-test

^a^ Measured at follow-up

### Parasitological status

The prevalence of STH infection in the overall sample was 8.1% at baseline, and this remained the same at follow-up. Less than 1% of the children had moderate to heavy STH infection. In all three countries, the STH prevalence at baseline and at follow-up did not significantly differ between intervention schools and control schools (Table 3). Hookworm accounted for more than 80% of the STH infection.

**Table 3:** Parasitological status, weight status and oral health status of children in intervention schools and control schools in Cambodia, Indonesia, Lao PDR and the pooled regional countries at baseline and follow-up

	Cambodia			Indonesia			Lao PDR			Regional (pooled)		
	FIT	Control	P*	FIT	Control	P*	FIT	Control	P*	FIT	Control	P*
	Parasitological status											
STH-prevalence at baseline *(n, %)*	22 (9.1)	18 (7.7)	0.573	3 (1.5)	6 (3.0)	0.298	25 (9.9)	37 (15.6)	0.059	50 (7.1)	61 (9.1)	0.195
STH-prevalence at follow-up *(n, %)*	34 (15.9)	14 (9.1)	0.056	2 (1.0)	6 (3.1)	0.120	22 (8.4)	25 (10.6)	0.419	58 (8.4)	45 (7.7)	0.630
	Weight status											
Prevalence of thinness at baseline *(n, %)*	21 (8.9)	23 (9.9)	0.718	15 (6.1)	25 (10.6)	0.076	16 (5.9)	19 (7.6)	0.434	52 (7.5)	67 (9.9)	0.112
Prevalence of thinness at follow-up *(n, %)*	31 (13.2)	36 (15.5)	0.485	14 (5.7)	23 (9.7)	0.095	25 (9.1)	21 (8.3)	0.748	70 (10.7)	80 (12.5)	0.294
	Oral health status (permanent dentition)^a^											
Dental caries prevalence at baseline *(n, %)*	32 (13.3)	43 (18.1)	0.149	26 (10.6)	23 (9.7)	0.741	36 (15.7)	40 (18.9)	0.382	94 (13.1)	106 (15.4)	0.223
Dental caries prevalence at follow-up *(n, %)*	126 (52.7)	137 (58.1)	0.243	75 (30.5)	83 (35.0)	0.288	68 (29.8)	80 (38.3)	0.062	269 (37.7)	300 (44.0)	*0.017*
Prevalence of PUFA at baseline *(n, %)*	1 (0.4)	3 (1.3)	0.370^1^	0 (0.0)	0 (0.0)	-	1 (0.4)	5 (2.4)	0.110^1^	2 (0.3)	8 (1.2)	0.060^1^
Prevalence of PUFA at follow-up *(n, %)*	17 (7.1)	23 (9.8)	0.302	21 (8.6)	18 (7.6)	0.704	10 (4.4)	16 (7.7)	0.149	48 (6.7)	57 (8.4)	0.265
DMFT at baseline *(mean ± sd)*	0.18 ± 0.51	0.30 ± 0.73	0.127	0.15 ± 0.50	0.13 ± 0.44	0.757	0.28 ± 0.78	0.37 ± 0.90	0.324	0.20 ± 0.61	0.26 ± 0.71	0.181
DMFT at follow-up *(mean ± sd)*	1.00 ± 1.20	1.29 ± 1.49	0.066	0.50 ± 0.92	0.59 ± 0.96	0.257	0.54 ± 1.06	0.79 ± 1.28	*0.026*	0.68 ± 1.09	0.89 ± 1.29	*0.003*
DMFT increment *(mean ± sd)*	0.82 ± 1.07	0.99 ± 1.30	0.262	0.35 ± 0.72	0.46 ± 0.79	0.168	0.26 ± 0.81	0.41 ± 1.11	0.373	0.48 ± 0.91	0.63 ± 1.12	*0.049*
PUFA at baseline *(mean ± sd)*	0.00 ± 0.06	0.02 ± 0.16	0.309	0.00 ± 0.00	0.00 ± 0.00	-	0.01 ± 0.13	0.02 ± 0.15	0.084	0.00 ± 0.08	0.01 ± 0.13	*0.049*
PUFA at follow-up *(mean ± sd)*	0.10 ± 0.37	0.13 ± 0.42	0.312	0.13 ± 0.44	0.09 ± 0.34	0.663	0.08 ± 0.47	0.11 ± 0.42	0.149	0.10 ± 0.43	0.11 ± 0.40	0.267
PUFA increment *(mean ± sd)*	0.09 ± 0.35	0.11 ± 0.38	0.483	0.13 ± 0.44	0.09 ± 0.34	0.663	0.07 ± 0.39	0.09 ± 0.41	0.434	0.10 ± 0.40	0.10 ± 0.38	0.548
Preventive fraction (DMFT) (*%*)	18.3%			22.4%			38.0%			23.9%		

χ^2^-test for dichotomous variables, Mann-Whitney U-test for continuous variables. ^1^ Fisher’s exact test for small numbers

^a^ 117 children excluded from analysis in Lao PDR because of an overlapping intervention of the Japan International Cooperation Agency

Respective missing values in Cambodia, Indonesia and Lao PDR: parasitological status (at baseline): 3, 76, 46, (at follow-up): 110, 78, 38; weight status (at baseline): 11, 3, 11, (at follow-up): 10, 2, 9; oral health status (at baseline): 1, 2, 6, (at follow-up): 3, 3, 10

Table 4 shows the factors that were significantly associated with STH infection at follow-up. Children with STH infection at baseline were nine times more likely to be (re-)infected at follow-up. The odds of STH infection were also higher for older children and children from larger families, while children who attended schools in urban areas had lower odds of STH infection. Every 10 % increase in the percentage of clean and functional toilets at school was associated with 0.91 (0.83; 1.00) lower odds of STH infection at follow-up. In other words, the odds of having STH infection were more than two times higher for children in schools with zero clean and functional toilets compared to children from schools where all toilets are fully clean and functional (1/(OR^10^) = 1/(0.91^10^)). The school level random effects variance and ICC show that 15.6% of the variance in STH infection at follow-up occurred between schools.

**Table 4:** Factors that are significantly associated with STH infection at follow-up in children in Cambodia, Indonesia and Lao PDR (pooled)^a^

	Model for parasitological status (*n* = 1162)	
	*OR (95% CI)*	*P* ^a^
	No STH infection at follow-up (reference) vs. STH infection at follow-up	
Child-level variables		
Age (years)	1.90 (1.19; 3.04)	0.007
Family size		
1 or no siblings (39.5%)	reference	
2 siblings (32.5%)	1.53 (0.83; 2.84)	0.175
3 or more siblings (28.0%)	2.06 (1.12; 3.80)	0.020
STH infection at baseline		
No (92.4%)	reference	
Yes (7.6%)	9.09 (4.98; 16.45)	<0.001
School-level variables		
Geographical location		
Rural (34.9%)	reference	
Urban (65.1%)	0.34 (0.18; 0.64)	0.001
Percentage of fully clean and functional toilets (per 10%)	0.91 (0.83; 1.00)	0.045
*Random effects*		
Country level variance (95% CI)	0.00 (0.00; 0.00)	ICC (%): 0.0
School level variance (95% CI)	0.78 (0.48; 1.26)	ICC (%): 15.6

Variables considered in the initial model: Child variables: FIT programme, age at follow-up, gender, number of siblings, STH infection at baseline, School variables: geographical location, number of enrolees at follow-up, number of water slots with water and soap, student to water slot ratio, percentage of clean and functional toilets

^a^Multilevel mixed-effects logistic regression

### Weight status

At baseline, the prevalence of thinness in the overall sample was 8.7% and this increased to 11.6% at follow-up. In Cambodia, both intervention and control schools showed a significant increase in the percentage of thin children between baseline and follow-up, but in Indonesia and Lao PDR the prevalence of thinness remained stable over the 2-year period. In all three countries, the prevalence of thinness did not significantly differ between intervention and control schools at baseline, nor at follow-up (Table 3).

The prevalence of overweight increased from 3.4% to 5.6% in Cambodia (*P* = 0.002), from 13.9% to 22.7% in Indonesia (*P* < 0.001) and from 5.5% to 8.8% in Lao PDR (*P* = 0.001).

Table 5 shows the factors that were significantly associated with thinness at follow-up. Children who were thin at baseline were 57 times more likely to remain thin at follow-up. The odds of being thin at follow-up were also higher for children who were stunted at baseline and for children with more decayed, missing and filled teeth (DMFT) at follow-up, while the odds were lower for children attending urban schools. The school- and country-level variance was close to zero, which indicates that thinness at follow-up was independent of the children’s school or country.

**Table 5:** Factors that are significantly associated with thinness at follow-up in children in Cambodia, Indonesia and Lao PDR (pooled)^a^

	Model for weight status (*n* = 1454)	
	*OR (95% CI)*	*P* ^a^
	Not thin at follow-up (reference) vs. Thin at follow-up	
Child-level variables		
Thin at baseline		
No (91.9%)	reference	
Yes (8.1%)	57.3 (34.5; 95.0)	<0.001
Stunted at baseline		
No (69.5%)	reference	
Yes (30.5%)	1.98 (1.27; 3.09)	0.003
DMFT at follow-up	1.28 (1.10; 1.50)	0.001
School-level variables		
Geographical location		
Rural (33.2%)	reference	
Urban (66.8%)	0.60 (0.38; 0.96)	0.032
*Random effects*		
Country level variance (95% CI)	0.00 (0.00; 0.00)	ICC (%): 0.0
School level variance (95% CI)	0.00 (0.00; 0.00)	ICC (%): 0.0

Variables considered in the initial model: Child variables: FIT programme, age at follow-up, gender, number of siblings, thin at baseline, stunted at baseline, DMFT at follow-up, School variables: geographical location, number of enrolees at follow-up, number of water slots with water and soap, student to water slot ratio, percentage of clean and functional toilets

^a^Multilevel mixed-effects logistic regression

### Oral health status

At baseline, 94.4% of the children in the overall sample had dental caries in the primary dentition (with a mean dmft of 9.2 ± 4.4) and 73.2% of them had odontogenic infections (with a mean pufa of 3.8 ± 2.6).

The baseline oral health status of the permanent dentition was comparable between children from intervention schools and control schools (Table 3). In all three countries, children in intervention schools had a lower prevalence of dental caries in the permanent dentition at follow-up and a lower increment in DMFT between baseline and follow-up in comparison to children in controls schools, although these were only statistically significant in the overall sample. The preventive fraction for DMFT was 23.9% in the overall sample, and 18.3%, 22.4%, 38.0% in Cambodia, Indonesia and Lao PDR, respectively (Table 3). There were no significant differences in the prevalence of odontogenic infections and PUFA increment between intervention and control schools.

Table 6 describes the factors that were significantly related with DMFT increment. The DMFT increment was significantly lower in intervention schools compared to control schools in the full model. Children who had more permanent teeth at baseline, younger children and children attending schools in urban areas had higher increment in DMFT between baseline and follow-up. The random effects variance and ICC show that 14.3% of the variance in DMFT increment occurred between schools and 9.9% between countries.

**Table 6:** Factors that are significantly associated with DMFT increment in children in Cambodia, Indonesia and Lao PDR (pooled)^a^

	Model for oral health status (*n* = 1395)	
	*β (95% CI)*	*P*
	DMFT increment	
FIT Programme		
No (48.9%)	reference	
Yes (51.1%)	−0.15 (−0.29; −0.01)	0.036
Child-level variables		
Age (years)	−0.12 (−0.23; −0.01)	0.04
Number of permanent teeth at baseline	0.04 (0.02; 0.06)	<0.001
School-level variables		
Geographical location		
Rural (35.4%)	reference	
Urban (64.6%)	0.39 (0.22; 0.57)	<0.001
*Random effects*		
Country level variance (95% CI)	0.10 (0.02; 0.56)	ICC (%): 10.1
School level variance (95% CI)	0.04 (0.02; 0.08)	ICC (%): 14.0

Variables considered in the initial model: Child variables: FIT programme, age at follow-up, gender, number of siblings, number of permanent teeth at baseline, School variables: geographical location, number of enrolees at follow-up, number of water slots with water and soap, student to water slot ratio, percentage of clean and functional toilets

^a^Multilevel mixed-effects linear regression

^b^117 children excluded from analysis because of an overlapping intervention of the Japan International Cooperation Agency in Lao PDR

## Discussion

WASH and school health programmes have strong potential to contribute to better health of children inlow and middle-income countries. The essence of the FIT programme lies in the institutionalization of a package of simple interventions within the education sector to establish hygiene habits and address some of the most prevalent childhood diseases in Southeast Asia. The FIT interventions – namely handwashing with soap, toothbrushing with fluoride toothpaste, biannual deworming and improved WASH infrastructure – are all underpinned by ample evidence for their respective effectiveness for improving child health in controlled settings [9, 25, 28–31]. Still, it is critical to conduct programme evaluation and impact research of such proven health and WASH interventions under real-life conditions where there is typically less or no control of possible cofounding factors. A government-run school programme, implemented by education staff without further external support provides such a real-life setting. Results of such research help to understand factors that facilitate the translation of the evidence from controlled settings into realistic public health promotion strategies, and, ideally, whether they can be applied at scale.

This study evaluated the 2-year effect of the FIT programme on parasitological, weight and oral health status in children attending elementary schools in Cambodia, Indonesia and Lao PDR participating in the Fit for School Programme. The study found that the FIT interventions significantly reduced the development of dental caries in children (by 24% at average). Due to specific circumstances that were not anticipated prior to the study, no significant decreases in the prevalence of STH infection and thinness were observed, which does not mean that the interventions did not work. The following paragraphs discuss this aspect in more detail.

### Discussion of parasitological results

The FIT programme was expected to improve children’s parasitological health via three mechanisms:^.^ biannual administration of albendazole or mebendazole is an efficacious method to treat existing worm infection [28], handwashing with soap interrupts transmission of helminthiases from contaminated soil or infected faeces [29] and improved access to WASH contributes to a reduction of helminthiases in the school environment [25, 32]. This was supported by findings from the former HOS in the Philippines, where the FIT programme led to a significant reduction in the prevalence of moderate to heavy STH infection [17]. However, this study did not show a similar effect.

The general prevalence of STH infection was surprisingly low at baseline (unlike in the Philippines), which did not leave much room for further improvement. The low baseline prevalence was unexpected in view of previously reported estimates of the parasitic disease burden in Southeast Asian countries [33], and could be indicative of effective implementation of the already existing national deworming programmes. Yet, it should be noted that the prevalence rates in this study are clearly not a representative reflection of national prevalence estimates, which revealed prevalences up to 86%, 90% and 66% in Cambodia, Indonesia and Lao PDR, respectively (with a mean prevalence of approximately 30%) [33], and that other geographical areas within the countries may show different results.

Interestingly, this study found that children with STH infection at baseline were nine times more likely to be re-infected at follow-up despite the mass drug administration scheme. This corresponds with previous studies reporting that anthelmintic drugs only provide a temporal reduction in morbidity, but do not prevent rapid reinfection [34]. This emphasises the need for complementary environmental STH control interventions, including improvement of sanitation (e.g. access to clean latrines and latrine maintenance) and interventions that promote hygiene habit formation in schools and in the home environment (e.g. handwashing with soap prior to eating and after defecation).

### Discussion of weight status results

This study did not find a programme effect on children’s weight status, which is not entirely surprising in view of the parasitological findings discussed above. Weight status was chosen as a relevant health indicator under the assumption that successful treatment of worm infection would enhance weight gain (“catch-up”). This was based on the Philippine HOS and other studies, where children’s BMI significantly increased after 1 year of regular deworming, possibly as a result of the substantial reduction in moderate to heavy worm infestation [17], although the actual evidence for this mechanism is not strongly supported by literature [35]. Circumstances in the current study were unexpectedly different, with less than 1% of children suffering from heavy worm infection at baseline and follow-up, so that no programme effect on parasitic health status was found.

The major causes of thinness are poor nutrition and infectious diseases, including diarrhoea. Handwashing with soap and safe sanitation are obvious measures to prevent infectious diseases, although the evidence of their effect on weight status and child growth is weak due to a lack of high quality randomised trials [36]. Literature suggests that interventions to promote hand hygiene, water and sanitation are *necessary*, but they do not have sufficient impact to address the chronic and persistent burden of underweight and thinness if they are not combined with efforts to tackle the root causes, including nutritional interventions and changes to the broader living environment [37]. Severe dental caries is another strong determinant of underweight [38–40], as also confirmed by the findings of this study, which suggests that prevention and treatment of dental decay should be considered among priority interventions addressing the burden of malnutrition.

Notably, data of this study suggest that obesity is on the rise in Southeast Asian countries, in particular in Indonesia. The emergence of obesity as a major public health problem in developing countries has been frequently reported [41, 42]. This paradoxical coexistence of both, childhood obesity and childhood undernutrition (also termed as the “double burden of disease”), will have important implications for the planning and redirection of health promotion strategies in the school context. A healthy nutrition environment at school (with nutrition choices low in sugar and fat), combined with regular physical activity, could be effective strategies to address obesity in school health programmes [43].

### Discussion of oral health results

The daily toothbrushing intervention of the FIT programme significantly contributed to the prevention of dental caries. The prevented fraction was 24% in the overall sample, ranging from 18% in Cambodia, 22% in Indonesia to 38% in Lao PDR. The findings are in concordance with findings from a Cochrane meta-analysis on the caries-preventive benefits of fluoride toothpaste in children [31]. The difference in prevented fraction between individual countries, might be attributed to implementation quality. Considering that the toothbrushing intervention was only performed once daily on schooldays, more dental caries may be prevented if the frequency of toothbrushing is increased to at least twice daily according to the recommendations of evidence-based guidelines [44].

The study results of all countries highlight that the burden of oral diseases in the Southeast Asian region is extremely high. More than 70% of children had severe dental infection, which can lead to eating and sleeping impacts, poor quality of life, school absenteeism and growth retardation [3, 37]. In order to reduce this neglected burden of oral diseases, full integration of oral health into public health promotion strategies and school health programmes is essential. The primary focus should be on preventing the disease via regular toothbrushing with fluoride toothpaste, since it is a proven, simple and realistic intervention that does not require involvement of health professionals. Most important is the development of regular habits for toothbrushing. Next level interventions may include additional fluoridation, such as the use of fluoride gel, and oral urgent treatment to address pain and suffer among the children [45].

### Challenges of real life implementation research and implications for future research

#### Limitations of the study design

Real-life implementation research brings along challenges in study design and the extent to which research conditions can be controlled, which should be considered in the interpretation of the study findings. First, randomisation of intervention and control schools was not possible. Intervention schools were purposively selected by the MoEs with the primary aim to test the FIT approach in a local context, and to create a country-specific model template for implementation and scale-up. The purposive sampling method may have introduced selection bias towards more favourable school conditions in intervention schools. The potential effect of this bias was minimized by assigning control schools with nearest location to the intervention schools, so that similar socioeconomic parameters could be assumed. However, this made the study more vulnerable to the effects of unplanned crossover or ‘spontaneous programme scale-up’ as, in fact, there were a few control schools that were implementing the FIT interventions on their own initiative. Secondly, an unplanned and previously un-reported overlapping oral treatment programme by Japanese International Cooperation Agency in Lao PDR interfered with the evaluation of the FIT programme, and therefore eight schools had to be excluded from the oral health analysis.

#### Challenges in implementation

The success of the FIT programme fully depends on the quality of programme delivery, which is built upon commitment and capacity of teaching staff, school leadership and local participation. No additional programme staff interacts with or supports the school staff on a daily basis. Even if interventions are proven highly effective, their full potential will not be reached if adherence to the protocol and implementation is poor. Although implementation quality was not assessed in the study, the data strongly suggest that there were big differences in implementation among schools, as illustrated by the example of oral health results. The mean DMFT increment in the overall intervention sample was 0.48 ± 0.91, however, there was wide variation between the intervention schools. In some intervention schools, the mean DMFT increment was close to zero (minimum: 0.07 ± 0.45), while in others the mean DMFT increased by more than 1 (maximum: 1.39 ± 1.20) (results not shown). The DMFT increment also greatly differed between countries, with Lao PDR having the lowest caries increase on average and Cambodia the highest (Table 3). All schools were supposed to implement the same intervention of once-daily toothbrushing with fluoride toothpaste, yet these results clearly show that DMFT increase greatly varied within schools and countries. This points to differences in programme compliance, since the interventions’ potential effect should in principle be similar irrespective of the setting, as long as the intervention is the same. Therefore, it is of utmost importance that school management structures and compliance with the protocol are considered in the implementation of the FIT programme or any other programme, particularly if health effects are to be achieved. Good implementation requires leadership within the education sector and relies on a collaborative effort between the local government, the school administration, teaching staff and the local community for which roles and responsibilities need to be clearly defined.

#### Choice of health indicators

STH infection, weight status and dental caries were chosen as key indicators for the health effect evaluation of the FIT programme, based on positive experiences from the former HOS in the Philippines [17] and available evidence. These are relevant health outcomes from a programme and advocacy perspective. However, in retrospect, these indicators also have limitations to evaluate the programme’s potential health effect, considering the previously described implementation challenges and the fact that these health outcomes are difficult to change within the study’s short time interval. Therefore, it is important that future programme evaluation research also explores options of including relevant intermediary health outcomes in addition to health indicators, such as hygiene behaviour change, rather than *solely* measuring conventional health indicators.

Healthy hygiene behaviours are key determinants of child health, and their benefits are most pronounced when they are practiced habitually on a regular basis. Successful and sustained incorporation of healthy hygiene habits in the lives of children would provide lifelong health benefits, which can even be transferred to their families, friends and communities. A valuable area for future research would therefore be to assess whether WinS and health programmes, including the FIT programme, are successful in the formation of children’s healthy hygiene habits.

## Conclusions

This study evaluated the two-year effect of the Fit for School programme on relevant health outcomes of children in Cambodia, Indonesia and Lao PDR. It found that the toothbrushing intervention significantly contributed to the prevention of dental caries in children. A clear asset of the study was that it describes real-life implementation research to assess whether a combination of relevant and already proven health and WASH interventions is effective in improving child health when delivered in schools as an integrated hygiene promotion package. Moreover, the results and their interpretation clearly highlight that effect evaluation research of WinS and health programmes encounters many challenges. These include restrictions in randomisation, the potential of crossover effects, challenges related to implementation quality, and unforeseen conditions that are beyond the researchers’ control, such as the interference of other health programmes. These challenges make it difficult to demonstrate the programme’s full potential effect, and this likely explains why no direct effect on weight status and STH infection was observed. Herewith, the study provides important learnings for future evaluation research, which points the way forward for also incorporating intermediary measures of behavioural outcomes and indicators of implementation quality, in addition to health indicators, in order to evaluate and understand how WinS programmes possibly lead to health benefits through implementation processes and their potential effect on hygiene habit formation.

The study suggests that even the most effective and simplest of health interventions, such as toothbrushing with fluoride toothpaste, handwashing with soap or deworming plausibly depend on implementation quality to reach their full beneficial potential. The traditional complexity of school health and WinS with multiple cross-sectoral roles and responsibilities calls for a governance and management simplification under the education sector’s leadership. As much as the education sector has been able to improve schooling rates and quality of education, it is overdue that ownership, governance and financing for school health and WinS are seen equally important for children’s health and education attainment. This includes regular monitoring and evaluation of WinS and School Health implementation quality through Education Management and Information Systems and other surveillance tools, as well as a supportive policy context. Schools as health-promoting settings can only be effective in achieving better hygiene behaviour, in providing preventive health or WASH services if they are able to manage, monitor and finance such services sustainably and consistently according to government guidelines. Achieving the ambitious targets of the SDGs in health, education and WASH will only be realistic with such a paradigm shift.

## Abbreviations

BMI: Body Mass Index
DMFT: Number of decayed, missing and filled permanent teeth
FIT: Fit for School
GIZ: German Development Cooperation
HOS: Health Outcome Study
ICC: Intraclass correlation
Lao PDR: Lao People’s Democratic Republic
MoE: Ministry of Education
MoH: Ministry of Health
PUFA: Number of teeth with pulp involvement, ulcerations, fistula and abscesses
SDG: Sustainable Development Goals
SEAMEO INNOTECH: South-East Asian Ministers of Education Organization Regional Centre for Educational Innovation and Technology
SES: Socioeconomic status
STH: Soil-transmitted helminth infection
WASH: Water, Sanitation and Hygiene
WHO: World Health Organization
WinS: WASH in Schools
